# Assessment of antibiotic prescribing patterns and antimicrobial resistance awareness among dentists: a mixed-methods study

**DOI:** 10.1017/ash.2026.10838

**Published:** 2026-09-29

**Authors:** Ridhi Ghodela, Naveen Kumar P.G., Mahesh R. Khairnar, Savitha Priyadarsini S, Tanya Thakkar, Neha Shukla, Unnati Gupta

**Affiliations:** Faculty of Dental Sciences, Banaras Hindu University, Varanasi, UP, India

## Abstract

**Background:**

Antimicrobial resistance is a global “silent pandemic” with dentistry contributing 10%–12% of outpatient antibiotic use. In low- and middle-income countries such as India, the absence of context-specific national dental antibiotic guidelines, widespread over-the-counter antibiotic access, and diagnostic limitations complicate rational prescribing and antimicrobial stewardship.

**Methods:**

A sequential explanatory mixed method study was conducted over 12 months in Varanasi. The quantitative component analyzed 2,844 dental prescriptions across primary, secondary, and tertiary care settings. Prescribing appropriateness was assessed using international evidence-based guidelines from the American Dental Association and Faculty of General dental Practice (UK). The qualitative component included 30 in-depth interviews with dental practitioners, analyzed using inductive thematic analysis to explore determinants of prescribing behavior.

**Results:**

Antibiotics were prescribed in 73.1% of cases, of which 69.6% were inappropriate. High prescribing rates observed in conditions where antibiotics are generally not indicated, including irreversible pulpitis (73.3%), apical periodontitis (92.3%), and implant procedures (100%). Broad-spectrum antibiotics predominated, particularly amoxicillin-clavulanic acid (43.0%) and cefpodoxime-clavulanic acid (21.7%). Qualitative findings identified that prescribing was driven by diagnostic uncertainty, defensive practice, patient expectations, and systemic constraints, including lack of national guidelines and unregulated antibiotic access.

**Conclusion:**

Inappropriate antibiotic prescribing in dentistry reflects a context-driven, multifactorial behavioral shaped by clinical, behavioral, and structural constraints rather than a simple knowledge deficit. Addressing this challenge requires context-specific national dental antibiotic guidelines, strengthened antimicrobial stewardship, improved diagnostic support, and regulatory enforcement. The proposed IDCARE+ framework offers a practical systems-based approach to promote rational antibiotic use in similar resource-constrained settings.

## Introduction

Antibiotics represent one of the most significant advances in modern medicine, markedly improving the prognosis of bacterial infections since the mid-20th century. By targeting essential microbial processes, these agents have reduced mortality and enabled complex medical and dental procedures, including maxillofacial surgeries and dental implants.^1^ However, their effectiveness is increasingly compromised by the rapid emergence of antimicrobial resistance (AMR), a situation largely driven by inappropriate and excessive use.^2^

The World Health Organization has identified AMR as a silent pandemic due to its substantial global impact. Bacterial resistance was directly responsible for 1.14 million of the 4.71 million deaths associated with AMR in 2021.^3^ Although hospital-based antibiotic use receives greater attention, outpatient settings contribute substantially to antibiotic consumption. Dentistry accounts for 10%–12% of outpatient antibiotic prescriptions, many for odontogenic conditions requiring definitive operative treatment.^4^ This issue is especially relevant in low-and middle-income settings such as India, where high antibiotic consumption, unregulated over-the-counter access and limited antimicrobial stewardship contribute to the escalation of resistance.^5^

In dental practice, antibiotics are prescribed for both therapeutic and prophylactic purposes. Therapeutic use is generally indicated as adjuncts to definitive dental treatment in patients with systemic involvement or risk of spreading infections, where prophylactic use is restricted to selected high-risk clinical situations. For most odontogenic infections, international guidelines from the American Dental Association (ADA) and the United Kingdom, recommend definitive local treatment.^6,7^ Despite this, broad-spectrum antibiotics are often prescribed empirically in minor procedures and localized conditions without clear indications.^8^

Inappropriate antibiotic use in dentistry significantly contributes to AMR, with studies reporting prescribing rates ranging from 27% to 88%, often involving broad-spectrum and non-recommended formulations.^9^ In the Indian context, the absence of context-specific national guidelines, along with reliance on prior training, peer practices and pharmaceutical influences, contributed to variability in prescribing behavior.

Existing research is largely based on self-reported prescribing practices and typically evaluates prescribing patterns or practitioner knowledge separately, limiting understanding of real-world clinical behavior and underlying drivers. This limitation is especially relevant in low- and middle-income settings such as India.

To address this gap, the present study employed a sequential explanatory mixed-methods design to (1) quantify therapeutic antibiotic prescribing patterns through prescription audits, (2) assess their appropriateness against evidence-based guidelines, and (3) explore the behavioral, clinical and systemic factors influencing prescribing practices among dental practitioners in Varanasi, India.

## Materials and methods

### Study design and ethical considerations

This study employed a sequential explanatory mixed-methods design, wherein a cross-sectional prescription audit was conducted to quantify antibiotic use, followed by a qualitative inquiry to explore underlying behavioral drivers. The quantitative phase adhered to the STROBE guidelines, and the qualitative phase followed the COREQ checklist. Ethical approval was obtained from the Institutional Ethics Committee (Ref: Dean/2024/EC/7369).

### Quantitative phase

#### Setting and participants

The audit was conducted in Varanasi, India, between January and December 2025. Prescriptions were collected from tertiary (academic), secondary (community health centers) and primary (public PHCs and private clinics) levels. Eligible records included all legible, signed dental prescriptions issued by registered dental practitioners, irrespective of antibiotic prescribing. Prescriptions lacking sufficient diagnostic information were excluded to maintain diagnostic–therapeutic concordance.

#### Data collection and sampling

Prescriptions were obtained using consecutive sampling during outpatient visits to capture maximum patient volume. Extracted variables included patient demographics, recorded clinical diagnosis and prescription details (drug name, dosage, frequency and duration). Potential sources of bias, including selection and information bias, were minimized through standardized data extraction and predefined inclusion criteria.

#### Assessment of prescribing appropriateness:

In absence of context-specific national guidelines in India, prescribing appropriateness was assessed against ADA and the Faculty of General Dental Practice (UK, FGDP) recommendations for therapeutic antibiotic prescribing. Prescriptions were classified as appropriate when aligned with guideline and inappropriate when antibiotics were prescribed for conditions requiring definitive local treatment or when discrepancies existed in dosage, frequency, or duration. To ensure reliability, subset of prescriptions was independently reviewed by two clinical experts, with disagreements resolved by a third reviewer. Inter-rater reliability demonstrated good internal consistency (Cronbach’s α = 0.80).

### Sample size and statistical analysis

#### Sample size

Sample size was calculated using an online calculator (https://www.raosoft.com/samplesize.html) assuming a 50% response distribution, 5% margin of error and 90% confidence level for an estimated annual outpatient population of 300,000, yielding a minimum sample of 2,382 prescriptions. A total of 2,844 prescriptions were analyzed using SPSS version 23. Quantitative variables were summarized using frequencies and percentages. As this was a descriptive study, no adjustment for confounding variables was performed.

### Qualitative phase: in-depth interviews

The qualitative component followed the thematic analysis framework by Braun and Clarke using an inductive approach within an interpretivist paradigm.^10^ A purposive sampling strategy with maximum variation was used to recruit dental practitioners across public, private and academic settings. Participants were approached face-to-face and invited to participate voluntarily.

Interviews were conducted by female (postgraduate student) trained researcher with prior experience in qualitative research. No prior relationship was established with participants, and were informed about the study purpose before interviews. Participants were assigned unique identification codes (eg, P1–P30), to maintain confidentiality. Participants characteristics are summarized in Table 1 and detailed characteristics in Supplementary Table S1.

**Table 1.** Demographic and professional characteristics of interview participants (N = 30) Table 1 long description.

Characteristics	Category	N (%)
**Gender**	Male	20 (66.7%)
	Female	10 (33.3%)
**Age (years)**	Mean ± SD	40.7 ± 8.3
**Highest qualification**	BDS	11 (36.7%)
	MDS	19 (63.3%)
**Primary practice setting**	Private practice	10 (33.3%)
	Government (PHC/CHC)	10 (33.3%)
	Teaching Institution	10 (33.3%)
**Years of clinical practice**	<5	8 (26.7%)
	5–10	13 (43.3%)
	>10	9 (30%)

BDS, Bachelor of Dental Surgery; MDS, Master of Dental Surgery; PHC, Primary Health Centre; CHC, Community Health Centre; SD, standard deviation.

#### Data collection

Data were collected through face-to-face semi-structured in-depth interviews (30–60 min) using a pilot-tested and iteratively refined interview guide focusing on AMR awareness, prescribing practices, patient expectations, and systemic constraints. Interviews were audio-recorded, supplemented with field notes and analyzed concurrently with data collection. Thematic saturation was achieved at the 26th interview and confirmed with additional interviews (n = 30).

#### Data analysis

Data was analyzed using inductive thematic content analysis. Transcripts were independently coded manually by three researchers, with discrepancies resolved through consensus to ensure reliability. An initial coding framework was developed iteratively from the data and refined into coding structure to organize emerging themes. Themes were developed inductively and supported by verbatim quotations. Trustworthiness was ensured through member checking, triangulation with quantitative findings, maintenance of an audit trail and reflexive journaling.

## Results

### Quantitative findings

A total of 2,844 dental prescriptions were analyzed, of which 2,079 (73.1%) included antibiotics. Based on ADA and FGDP guideline recommendations, 865 prescriptions (30.4%) were classified as appropriate, while 1,979 (69.6%) as inappropriate. Most prescribed antibiotics are amoxicillin–clavulanic acid (43.0%) and cefpodoxime–clavulanic acid (21.7%) together accounting for 64.7% of all prescriptions. Other commonly prescribed agents included metronidazole (15.3%), ofloxacin–ornidazole (7.9%), cefuroxime–clavulanic acid (6.4%), and cefixime (5.5%).

In endodontic conditions, antibiotics were prescribed in 75.3% of cases (n = 1057/1404), despite operative management being the standard of care. High prescribing rates were observed in radicular cyst (92.9%), apical periodontitis (92.3%), periapical abscess (88.2%), and irreversible pulpitis (73.3%). In periodontal conditions, antibiotics were prescribed in 77.2% of cases (n = 560/725). High prescribing rates were observed in periodontal abscesses (100%), pericoronitis (99.5%), localized (73.7%), and generalized periodontitis (69.6%). In surgical and operative conditions, antibiotic was prescribed in 97.0% of cases (n = 293/302), Prescriptions were observed in all cases of dental implants, jaw fractures, and impactions. In oral mucosal, non-bacterial and functional conditions, antibiotics were prescribed in 40.9% of cases (n = 169/413). Notable use was observed in aphthous ulcers (61.7%), neuropathy (58.6%), and temporomandibular joint arthralgia (41.7%), whereas no antibiotic use was recorded in candidiasis and xerostomia. Prescribing appropriateness across clinical conditions in comparison with ADA and FGDP recommendations is presented in Table 2.

**Table 2.** Antibiotic prescribing patterns across dental diagnoses and comparison with guideline recommendations Table 2 long description.

Diagnosis	Total cases	Antibiotic prescribed n (%)	Antibiotic not prescribed n (%)	FGDP guideline	ADA guideline
**Pulpal and periapical conditions:**					
Reversible pulpitis	155	0 (0.0%)	155 (100%)	Not indicated	Not indicated
Irreversible pulpitis	434	318 (73.3%)	116 (26.7%)	Not indicated	Not indicated
Apical periodontitis	456	421 (92.3%)	35 (7.7%)	Not indicated	Not indicated
Radicular cyst	28	26 (92.9%)	2 (7.1%)	Not indicated	Not indicated
Periapical abscess	331	292 (88.2%)	39 (11.8%)	Not indicated	Not indicated
Total	1,404	1,057 (75.28%)	347 (24.72%)		
**Periodontal conditions:**					
Localised gingivitis	114	53 (46.4%)	61 (53.6%)	Not indicated	Not indicated
Generalised chronic gingivitis	109	58 (53.2%)	51 (46.8%)	Not indicated	Not indicated
Generalised periodontitis	122	85 (69.6%)	37 (30.4%)	Not indicated	Not indicated
Localised periodontitis	57	42 (73.7%)	15 (26.3%)	Not indicated	Not indicated
Periodontal abscess	116	116 (100%)	0 (0.0%)	Not indicated	**Indicated**
Pericoronitis	207	206 (99.5%)	1 (0.5%)	Not indicated	Not indicated
Total	725	560 (77.24%)	165 (22.76%)		
**Surgical and operative conditions:**					
Impaction	59	59 (100%)	0 (0.0%)	Not indicated	Not indicated
Orthodontic extraction	52	45 (86.5%)	7 (13.5%)	Not indicated	Not indicated
Oroantral fistula	46	46 (100%)	0 (0.0%)	**Indicated**	**Indicated**
Implant	56	56 (100%)	0 (0.0%)	Not indicated	Not indicated
Tooth fracture	35	33 (94.3%)	2 (5.7%)	Not indicated	Not indicated
Jaw fracture	54	54 (100%)	0 (0.0%)	**Indicated**	**Indicated**
Total	302	293 (97.01)	9 (2.98%)		
**Others conditions**					
Others	363	169 (46.5%)	194 (53.44)	Not indicated	Not indicated

FGDP, Faculty of General Dental Practice (UK); ADA, American Dental Association

### Qualitative finding

Four interrelated themes with corresponding subthemes were identified, reflecting antibiotic prescribing as a multifactorial, context-driven behavior.

### Theme 1: The Knowledge—Practice interface

Participants demonstrated awareness of AMR; however, this understanding was largely superficial and externalized, remaining experiential rather than grounded in formal microbiological knowledge. This resulted in a disconnect between awareness and clinical practice.

**1.1 Experiential Perception of Resistance:**

AMR was primarily recognized through clinical outcomes, such as delayed healing

*AMR means that the antibiotic that we are giving, the specific bacteria on which it was working earlier, now it will not work. We know when the patient is not getting relief, the swelling is not reducing, there is slow healing or persistent infection. (P2)*

*If you conduct a bacterial culture test in a normal individual, you will find that nearly 90% of the antibiotics show resistance. This is not just about one patient or one prescription—it shows how serious the problem become at the community level. (P8)*

**1.2 Diffusion of Responsibility**

Responsibility for AMR was often attributed to patient behavior and prescribing practices in other healthcare sectors.

*Most patients do not come in the beginning, first they will try clove or some powder then they will take antibiotics on their own, when they come there is swelling and systemic involvement so we prescribe antibiotics first (P18)*

*I am working in a teaching institute…. I have seen many cases where three or four antibiotics are written So, AMR is not happening only because of dentists; it is equally by the medical field as well. (P13)*

**1.3 Habitual Normalization and Educational Gaps**

Limited training and lack of education led to reliance on peer norms, resulting in habitual prescribing behaviors.

*When we pass out, there is no proper training on how to prescribe antibiotics. We just follow what seniors are prescribing or what medical representatives suggest. After some years, it becomes routine and then changing that practice becomes very difficult. (P15)*

*We do not have CMEs frequently, very rarely they are related to AMR or proper antibiotic prescribing. There are also hardly any workshops or seminars focused on this. Regular updates are needed because resistance patterns keep changing. (P4)*

### Theme 2: Prescribing as a “Safety-measure”

Antibiotic prescribing was frequently described as a precautionary strategy. In the presence of diagnostic uncertainty, resource limitations, and perceived clinical risk, antibiotics were used as compensatory tools to manage uncertainty and prevent adverse outcomes.

**2.1 Diagnostic Ambiguity and Resource Constraints:**

Limited diagnostic infrastructure and time constraints led clinicians to prescribe antibiotics.

*We don’t have Culture sensitivity kit, limited access to diagnostic tools further restricted evidence-based decision making and even patients are not ready to wait and afford, so we end up giving antibiotics. (P11)*

*If you have 500 dentists practicing, there will be few who are doing culture sensitivity test, we don’t have instruments or facilities, report comes after two weeks or sometimes not at all. (P24)*

**2.2 Risk Avoidance and Defensive Practice:**

Fear of complications, medico-legal concerns, and poor patient compliance reinforced defensive prescribing behavior. Clinicians described using antibiotics “to be on the safe side,” particularly in patients with irregular follow-up or poor oral hygiene.

*In India, we cannot always rely on patient’s oral hygiene, many patients do not follow oral hygiene instructions. They may consume tobacco or fail to brush properly. (P23)*

*We don’t want complications later… so we prescribe antibiotics. (P19)*

Together, these findings illustrate a shift toward “cautious medicine,” where prescribing decisions are guided by risk avoidance rather than strict adherence to clinical guidelines.

### Theme 3: Socio-Cultural influences on Antibiotic Prescribing

Antibiotic prescribing was influenced by patient expectations peer norms and social pressure.

**3.1 “Prescription as Satisfaction” Trap:**

Patients commonly associate effective care with antibiotics and prioritized immediate pain relief.

*Most of the patients come with the mindset that if pain is there, antibiotic is compulsory. They only want tablet. If we don’t prescribe, they feel dissatisfied. (P14)*

*When we try to explain that antibiotics are not required, patients feel we are wasting time (P13)*

In many cases, antibiotics were used as substitutes for procedures such as extraction or root canal treatment, reflecting operative avoidance and demand-driven prescribing.

**3.2 Normalization and Influencing Norms:**

Peer practices, pharmaceutical influence and digital media, contributing to the normalization of antibiotic use.

*When you see that everyone around you is prescribing antibiotics for similar cases, it becomes a normal thing. (P20)*

Additionally, patient exposure to online information led to demand for specific antibiotics,

*Patients get information from Google, YouTube and social media and they come asking for specific antibiotics. (P17)*

Together, these findings indicate that prescribing decisions are often compromised rather than evidence-based, influenced by interpersonal dynamics and social expectations. Antibiotics thus serve both clinical and symbolic roles.

### Theme 4: System-Level Barriers and the Need for Stewardship Reform

Structural and policy-level factors played a critical role in shaping prescribing behavior. These findings highlighted that inappropriate antibiotic use is not solely an individual decision but a system-level adaptation to contextual and regulatory constraints.

**4.1 The “Guideline Gap”:**

Absence of context-specific national guidelines, limiting adherence to international recommendations.

*Foreign guidelines are made for their own population. We cannot apply those guidelines directly to Indian patients. (P18)*

*If there were clear Indian guidelines, we could confidently avoid unnecessary antibiotics. But since there is no proper direction, many times antibiotics are given as a safer side. (P22)*

**4.2 Unregulated Access and Regulatory Gaps:**

Unregulated over-the-counter availability contributed to widespread antibiotics misuse.

*In India, buying antibiotics becomes like buying items from a grocery store, people go to the pharmacy and get antibiotics without any prescription. (P08)*

Although policies exist, their implementation remains inconsistent, contributing to normalization of antibiotic use at the community level.

**4.3 Readiness for Stewardship and Reform:**

Participants acknowledged overuse and expressed willingness for change.

*We are definitely overusing antibiotics. If this continues, then in the future even simple infections will not respond. (P29)*

**Figure 1.**
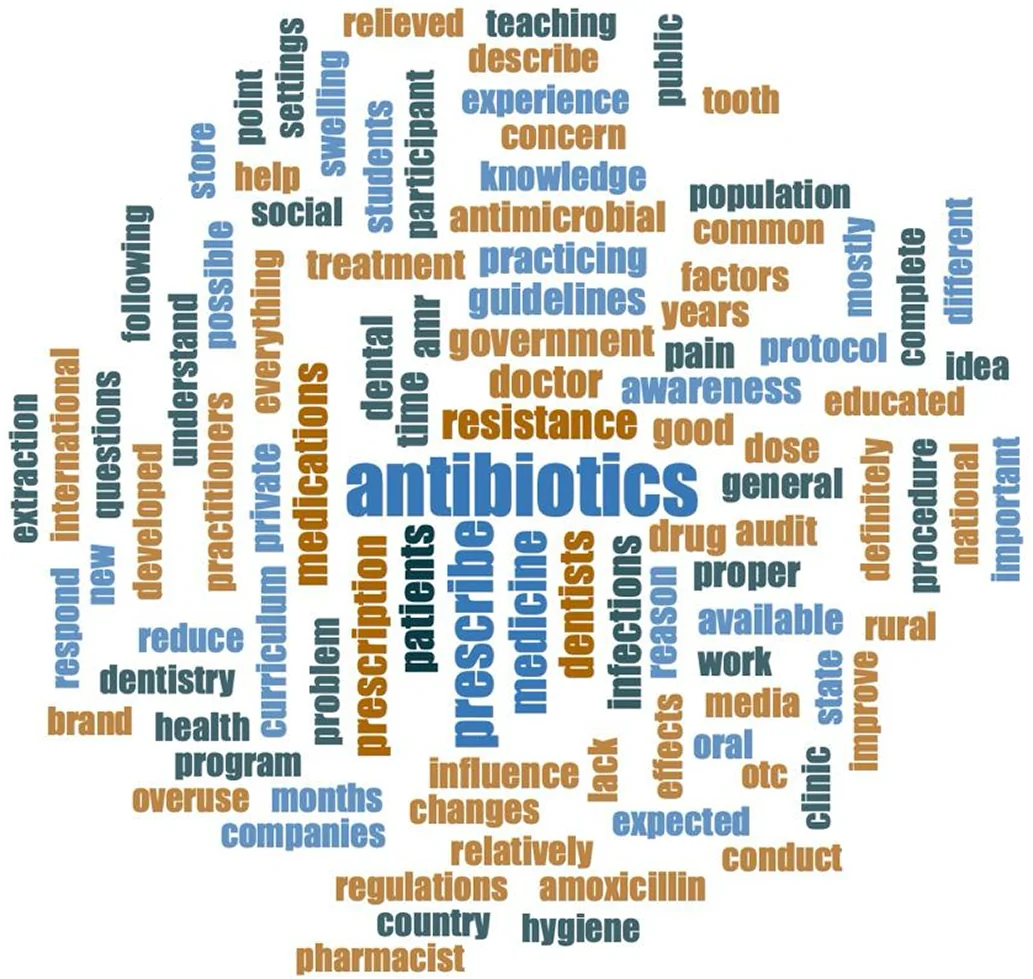
Word cloud visualizing dominant concepts identified in participant narratives.

**Figure 2.**
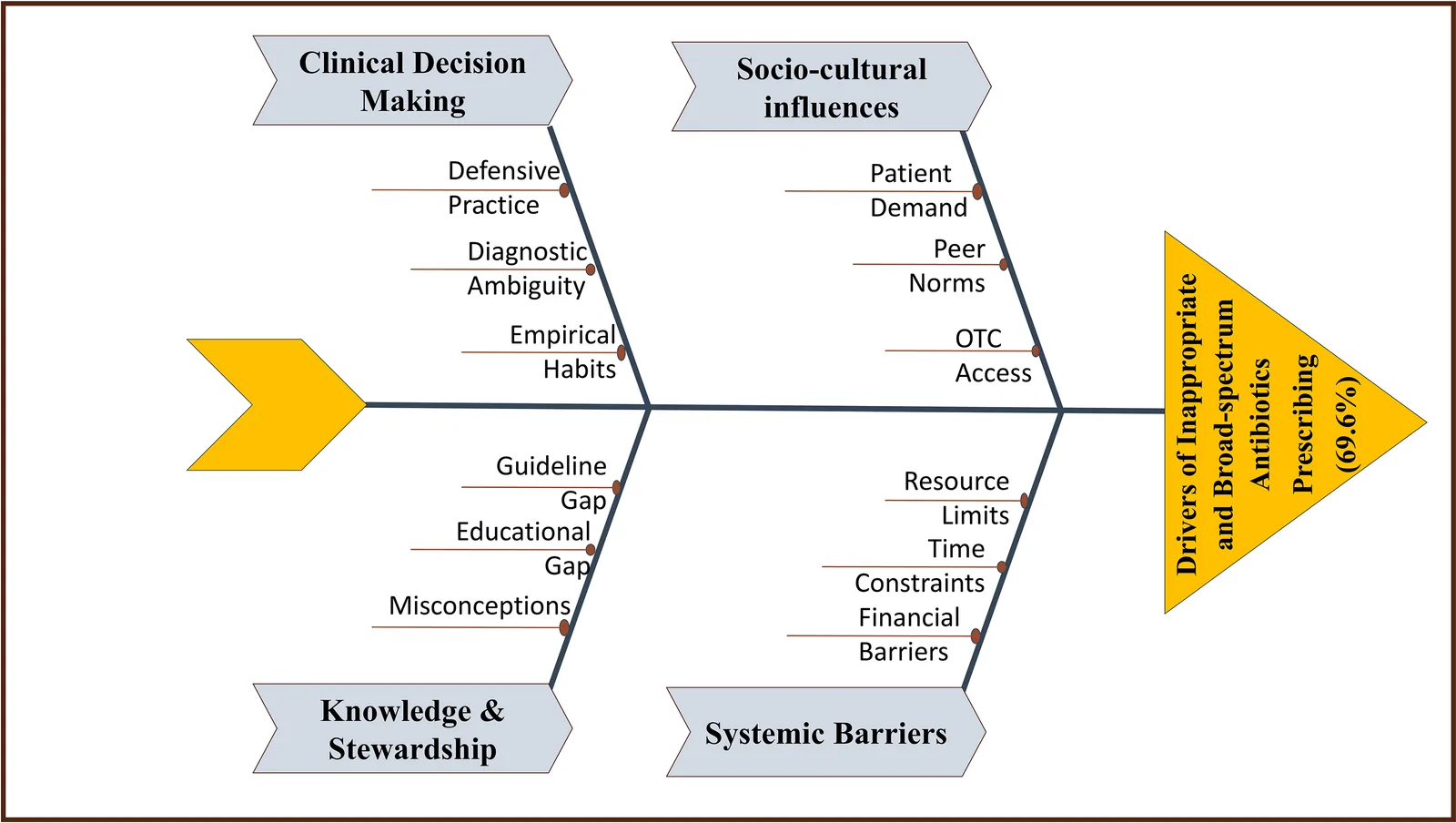
Fishbone diagram identifies multiple factors contributing to inappropriate antibiotic prescribing in dental practice, grouped into four key domains.

Illustrative verbatim quotations supporting each theme were provided in supplementary table S2. A word cloud was generated using NVivo, visually represents the most frequently occurring terms within participant narratives (Figure 1). Findings were further synthesized into an Ishikawa (fishbone) diagram to illustrate the root causes of inappropriate antibiotic prescribing (Figure 2),^11^ where the “head” represents inappropriate prescribing and the “bones” capture key drivers. Table 3 demonstrates the integration of quantitative and qualitative findings.

**Table 3.** Integrated quantitative–qualitative synthesis of antibiotic prescribing behavior Table 3 long description.

No.	Domain	Quantitative finding (Key result)	Qualitative driver	Integrated interpretation
**1**	**Over prescribing**	High antibiotic use (73.1%)	Defensive practice	Reflects risk-averse prescribing under uncertainty
**2**	**Endodontic conditions**	Antibiotics (75.3%) despite operative indication	Diagnostic ambiguity	Used as substitutes when definitive care is delayed
**3**	**Periodontal conditions**	Routine antibiotic use (77.2%)	Habitual norms	Driven by entrenched practices rather than evidence
**4**	**Surgical or prophylactic use**	Near-universal antibiotic use (97%)	Risk avoidance	Used as preventive safeguard against complications
**5**	**Nonbacterial or functional conditions**	Antibiotic use without infection (40.9%)	Patient expectations	Used for reassurance rather than necessity
**6**	**Drug selection pattern**	Broad-spectrum predominance including Amoxicillin-clavulanic acid (43%) and cefpodoxime-clavulanic acid (21.7%)	Empirical practice	Reflects lack of diagnostic support

**Figure 3.**
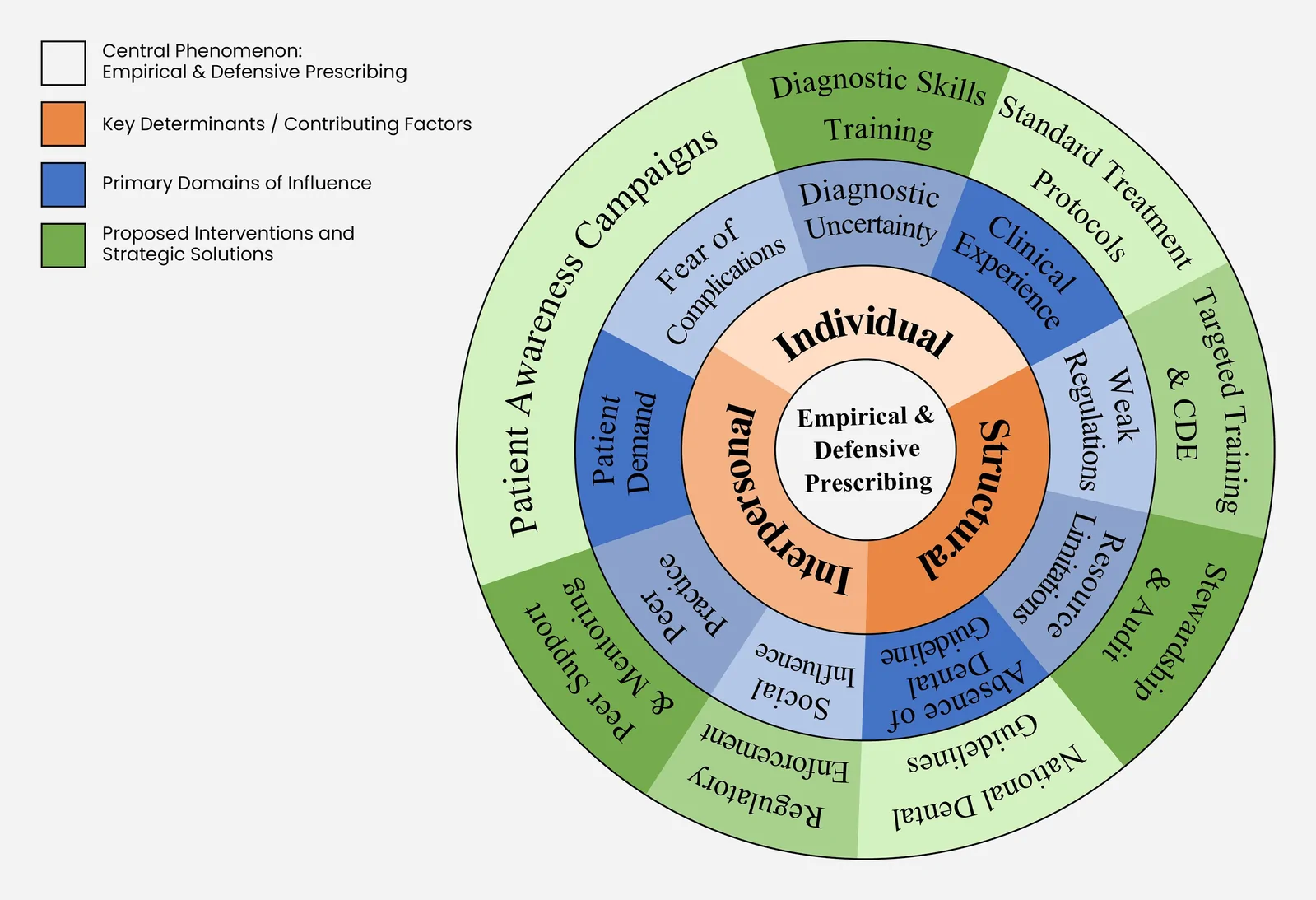
Figure 3 long description. Proposed ID-CARE+ Framework (Integrated dental contextual antibiotic ecosystem plus) for improving antibiotic stewardship in dentistry.

Based on these findings, the IDCARE+ framework (Figure 3) is proposed as a conceptual model to understand antibiotic prescribing as a dynamic system influenced by empirical and defensive practices. The “PLUS” layer—comprising Guidelines, Diagnostics, Stewardship, and Regulation—outlines potential intervention pathways to address the identified gaps and promote rational antibiotic use.

## Discussion

Through a sequential explanatory mixed-methods design, this study provides a comprehensive understanding of antibiotic prescribing behavior in dental practice within the Indian context. The findings demonstrate a high overall prescribing rate (73.1%), with 69.6% of prescriptions classified as inappropriate, highlighting substantial deviation from evidence-based recommendations. The integration of quantitative and qualitative data indicates that over-prescribing is not merely a result of inadequate knowledge, but reflects a context-driven, defensive adaptation to complex clinical realities. These include delayed patient presentation, high disease burden and the absence of context-specific national guidelines, limiting the direct applicability of international recommendations.

High prescription was observed in endodontic conditions (apical periodontitis: 92.3%; irreversible pulpitis: 73.3%) is explained by diagnostic ambiguity and defensive decision-making, despite clear recommendations favoring operative management. Similar patterns of excessive antibiotics prescribing have been reported in previous studies, with rates of 68.5% and 89%, respectively.^12,13^ This reflects a knowledge–practice gap, where antibiotics are used as a temporary “Band-Aid” measure when definitive care delayed due to systemic or logistical constraints. Evidence from other study supports that antibiotics offer minimal benefit when appropriate operative treatment is performed.^8^

Similarly, the high prescription observed in periodontal conditions (periodontitis: 69.6%–73.7%; gingivitis: 46.4%–53.2%) may reflect habitual normalization and peer-influenced clinical practices. Although antibiotics may be indicated for periodontal abscesses with systemic involvement, the consistently high prescribing observed for gingivitis and periodontitis, suggests routine use beyond evidence-based recommendations, which prioritize mechanical therapy. Widespread antibiotic prescribing has also been reported in the absence of systemic involvement.^14,15^ Other studies have similarly demonstrated reliance on personal clinical experience rather than standardized prescribing guidelines.^16,17^

In Surgical and operative conditions, antibiotics were prescribed in 97% of cases. Although antibiotics may be indicated for selected surgical procedures and high-risk patients, the high prescribing frequency suggests a precautionary approach extending beyond current evidence-based recommendations. Qualitative findings indicated that fear of postoperative complications and medico-legal concerns were key drivers of prescribing. Randomized trials have demonstrated limited benefits of routine antibiotics prophylaxis for uncomplicated dental procedures.^18,19^

The observed antibiotic use in nonbacterial and functional conditions (40.9%), including neuropathy (58.6%) and aphthous ulcer (61.7%), is explained by socio-cultural and interpersonal pressures. Prescribing in these cases reflects a negotiated clinical process, where antibiotics are used to meet patient expectations and provide reassurance rather than address infection. This finding aligns with the role of patient expectations and satisfaction in influencing prescribing behavior.^20^

The predominance of broad-spectrum antibiotics, particularly amoxicillin–clavulanic acid (43.04%) and cefpodoxime–clavulanic acid (21.74%) corresponds to the empirical prescribing patterns observed in the qualitative analysis. This reflects the absence of microbiological diagnostic support and reliance on empirical decision-making. Similar trends have been reported globally, indicating a tendency toward broad-spectrum antibiotic use in the absence of culture-based guidance.^15,21,22^

These prescribing behaviors are further shaped by systemic and structural constraints, including high patient load, limited consultation time, inadequate diagnostic infrastructure and easy OTC access to antibiotics. In such settings, antibiotics function as compensatory strategies when immediate definitive care is not feasible. Importantly, although practitioners demonstrated awareness of AMR, this awareness remained superficial and was often attributed to other healthcare sectors. This highlights a critical knowledge-action gap, where awareness does not translate into rational prescribing. Similar observations have been reported, indicating that knowledge alone is insufficient to drive behavioral change.^23,24^

Antibiotic prescribing is a context-driven, multifactorial socio-clinical behavior shaped by clinical uncertainty, habitual practices, patient expectations, sociocultural influences, and systemic constraints rather than a simple knowledge deficit. While these unsustainable practices contribute significantly to AMR in the India, combating them requires shifting from individual education to system-level strategies. The proposed IDCARE + framework addresses this by situating prescribing within individual, interpersonal, and structural determinants, utilizing its “PLUS” layer encompassing Guidelines, Diagnostics, Stewardship and Regulation target these barriers and promote rational use. Consequently, dentists should prioritize definitive operative treatments and reserve antibiotics for patients with systemic involvement or other evidence-based indications.

However, these findings are bounded by limitations: the study’s specific geographic focus may affect generalizability, qualitative insights rely on self-reported experiences and may subject to response bias. Prescribing appropriateness was assessed against international guidelines because India lacks context-specific national dental prescribing guidelines. In addition, the audit was limited to written dental prescriptions and didn’t capture antibiotics obtained without a written prescription.

## Supporting information

10.1017/ash.2026.10838.sm001 Ghodela et al. supplementary material Ghodela et al. supplementary material

## Data Availability

Supporting data associated with this study are available in the Fig share repository at https://doi.org/10.6084/m9.figshare.32221215.

## Supplementary material

The supplementary material for this article can be found at: https://doi.org/10.1017/ash.2026.10838.

## Table 1. Long description

A table with five rows and four columns summarizing the demographic and professional characteristics of 30 interview participants. The columns are labeled Characteristics, Category, and N (%). The rows detail gender distribution with 20 males (66.7%) and 10 females (33.3%). The mean age is 40.7 years with a standard deviation of 8.3 years. Highest qualifications include BDS with 11 participants (36.7%) and MDS with 19 participants (63.3%). Primary practice settings are evenly distributed with 10 participants each in private practice, government (PHC/CHC), and teaching institutions (33.3% each). Years of clinical practice are categorized as less than 5 years (8 participants, 26.7%), 5-10 years (13 participants, 43.3%), and more than 10 years (9 participants, 30%).

Navigate back to Table 1..

## Table 2. Long description

A table comparing antibiotic prescription rates across different dental diagnoses and guidelines. The table has 14 rows and 6 columns. Column headers are Diagnosis, Total cases, Antibiotic prescribed n (%), Antibiotic not prescribed n (%), FGDP guideline, and ADA guideline. Row labels include various dental conditions grouped under Pulpal and periapical conditions, Periodontal conditions, Surgical and operative conditions, and Others conditions. Each row lists the total cases, number and percentage of cases where antibiotics were prescribed, number and percentage of cases where antibiotics were not prescribed, and the guidelines from FGDP and ADA. Notable trends include high antibiotic prescription rates in conditions like apical periodontitis, periapical abscess, periodontal abscess, and pericoronitis, with varying adherence to FGDP and ADA guidelines.

Navigate back to Table 2..

## Table 3. Long description

A table with six rows and four columns. The columns are labeled Domain, Quantitative finding (Key result), Qualitative driver, and Integrated interpretation. Row 1: Over prescribing, High antibiotic use (73.1 percent), Defensive practice, Reflects risk-averse prescribing under uncertainty. Row 2: Endodontic conditions, Antibiotics (75.3 percent) despite operative indication, Diagnostic ambiguity, Used as substitutes when definitive care is delayed. Row 3: Periodontal conditions, Routine antibiotic use (77.2 percent), Habitual norms, Driven by entrenched practices rather than evidence. Row 4: Surgical or prophylactic use, Near-universal antibiotic use (97 percent), Risk avoidance, Used as preventive safeguard against complications. Row 5: Nonbacterial or functional conditions, Antibiotic use without infection (40.9 percent), Patient expectations, Used for reassurance rather than necessity. Row 6: Drug selection pattern, Broad-spectrum predominance including Amoxicillin-clavulanic acid (43 percent) and cefpodoxime-clavulanic acid (21.7 percent), Empirical practice, Reflects lack of diagnostic support.

Navigate back to Table 3..

## Figure 3. Long description

A diagram illustrating factors influencing empirical and defensive prescribing in dentistry. The central phenomenon is empirical and defensive prescribing, surrounded by key determinants and contributing factors. These factors are divided into individual, interpersonal, and structural categories. Individual factors include diagnostic uncertainty and fear of complications. Interpersonal factors encompass patient demand, peer influence, and social enforcement. Structural factors involve weak regulations, resource limitations, and the absence of dental guidelines. Proposed interventions and strategic solutions are also highlighted, including patient awareness campaigns, targeted training, stewardship, and regulatory enforcement. The diagram shows the relationships and interactions between these factors and solutions.

Navigate back to Figure 3..

## References

[1] Halawa EM , Fadel M , Al-Rabia MW , et al. Antibiotic action and resistance: updated review of mechanisms, spread, influencing factors, and alternative approaches for combating resistance. Front Pharmacol 2024;14:1305294. doi: 10.3389/fphar.2023.1305294. PMC10820715 38283841

[2] Salam MdA , Al-Amin MdY , Salam MT , et al. Antimicrobial resistance: a growing serious threat for global public health. Healthcare (Basel) 2023;11:1946. doi: 10.3390/healthcare11131946. PMC10340576 37444780

[3] Naghavi M , Vollset SE , Ikuta KS , et al. Global burden of bacterial antimicrobial resistance 1990–2021: a systematic analysis with forecasts to 2050. Lancet 2024;404:1199–1226. doi: 10.1016/S0140-6736(24)01867-1. PMC11718157 39299261

[4] Huynh CVT , Gouin KA , Hicks LA , et al. Outpatient antibiotic prescribing by general dentists in the United States from 2018 through 2022. J Am Dent Assoc 2025;156:382–389.e2. doi: 10.1016/j.adaj.2024.12.003. PMC12058375 40047737

[5] Laxminarayan R , Chaudhury RR. Antibiotic resistance in India: drivers and opportunities for action. PLoS Med 2016;13:e1001974. doi: 10.1371/journal.pmed.1001974. PMC4775002 26934098

[6] Lockhart PB , Tampi MP , Abt E , et al. Evidence-based clinical practice guideline on antibiotic use for the urgent management of pulpal- and periapical-related dental pain and intraoral swelling. J Am Dent Assoc 2019;150:906–921.e12. doi: 10.1016/j.adaj.2019.08.020. PMC8270006 31668170

[7] Palmer N. Antimicrobial Prescribing in Dentistry: Good Practice Guidelines, 3rd edition. London: Faculty of General Dental Practice (UK); Faculty of Dental Surgery, Royal College of Surgeons of England; 2020. https://cgdent.uk/antimicrobial-prescribing-indentistry/.

[8] Cope AL , Chestnutt IG , Wood F , et al. Dental consultations in UK general practice and antibiotic prescribing rates: a retrospective cohort study. Br J Gen Pract 2016;66:e329–e336. doi: 10.3399/bjgp16X684757. PMC4838445 27025554

[9] Stein K , Farmer J , Singhal S , et al. The use and misuse of antibiotics in dentistry. J Am Dent Assoc 2018;149;869–884.e5. doi: 10.1016/j.adaj.2018.05.034. 30261952

[10] Braun V , Clarke V. Using thematic analysis in psychology. Qual Res Psychol 2006;3:77–101. doi: 10.1191/1478088706qp063oa.

[11] Seong SM , Oh H , Park JS , et al. Incidence and root cause analysis of near-miss events in medical device use errors in intensive care units using Ishikawa diagram. Jpn J Nurs Sci 2025;22:e70024. doi: 10.1111/jjns.70024. PMC12434650 40948243

[12] Kumar KP , Kaushik M , Kumar PU , et al. Antibiotic prescribing habits of dental surgeons in Hyderabad City, India, for pulpal and periapical pathologies: a survey. Adv Pharmacol Sci 2013;2013:537385. doi: 10.1155/2013/537385. PMC3804281 24187549

[13] Teklay G , Tefera H. Appropriateness of antibiotic prescribing among patients treated for dental diseases in Mekelle city, Northern Ethiopia: a cross sectional study. J Health Popul Nutr 2024;43:153. doi: 10.1186/s41043-024-00636-x. PMC11439215 39342399

[14] Salako NO , Rotimi VO , Adib SM , et al. Pattern of antibiotic prescription in the management of oral diseases among dentists in Kuwait. J Dent 2004;32:503–509. doi: 10.1016/j.jdent.2004.04.001. 15304295

[15] Verma RK , Suresan V , Das S , et al. Antibiotic prescribing practices amongst the dental practitioners of Bhubaneswar City: a cross-sectional study. World J Dent 2022;13:520–526. doi: 10.5005/jp-journals-10015-2069.

[16] Dar-Odeh N , Abu-Hammad O , Al-Omiri M , et al. Antibiotic prescribing practices by dentists: a review. Ther Clin Risk Manag 2010;6:301–306. doi: 10.2147/TCRM.S9736. PMC2909496 20668712

[17] Dormoy J , Vuillemin MO , Rossi S , et al. Perceptions of antibiotic use and resistance: are antibiotics the dentists’ anxiolytics? Antibiotics (Basel) 2021;10:735. doi: 10.3390/antibiotics10060735. PMC8235224 34204526

[18] Momand P , Becktor JP , Naimi-Akbar A , et al. Effect of antibiotic prophylaxis in dental implant surgery: a multicenter placebo-controlled double-blinded randomized clinical trial. Clin Implant Dent Relat Res 2022;24:116–124. doi: 10.1111/cid.13068. PMC9306815 35075765

[19] Böhmer F , Hornung A , Burmeister U , et al. Factors, perceptions and beliefs associated with inappropriate antibiotic prescribing in German primary dental care: a qualitative study. Antibiotics (Basel) 2021;10:987. doi: 10.3390/antibiotics10080987. PMC8389002 34439037

[20] Thompson W , Tonkin-Crine S , Pavitt SH , et al. Factors associated with antibiotic prescribing for adults with acute conditions: an umbrella review across primary care and a systematic review focusing on primary dental care. J Antimicrob Chemother 2019;74:2139–2152. doi:10.1093/jac/dkz152. PMC6640312 31002336

[21] Alzahrani AAH , Alzahrani MSA , Aldannish BH , et al. Inappropriate dental antibiotic prescriptions: potential driver of the antimicrobial resistance in Albaha Region, Saudi Arabia. Risk Manag Healthc Policy 2020;13:175–182. doi:10.2147/RMHP.S247184. PMC7062393 32184688

[22] Farkaš M , Ivančić Jokić N , Mavrinac M , et al. Antibiotic prescribing habits and antimicrobial resistance awareness of dental practitioners in Primorsko-Goranska County, Croatia. Microb Drug Resist 2021;27:1482–1488. doi: 10.1089/mdr.2020.0478. 33913770

[23] Bhuvaraghan A , King R , Larvin H , et al. Antibiotic use and misuse in dentistry in India—a systematic review. Antibiotics (Basel) 2021;10:1459. doi: 10.3390/antibiotics10121459. PMC8698453 34943671

[24] Zetts RM , Stoesz A , Garcia AM , et al. Primary care physicians’ attitudes and perceptions towards antibiotic resistance and outpatient antibiotic stewardship in the USA: a qualitative study. BMJ Open 2020;10:e034983. doi: 10.1136/bmjopen-2019034983. PMC7365421 32665343

