# Supplementary material for: Assessment of antibiotic prescribing patterns and antimicrobial resistance awareness among dentists: a mixed-methods study
Source: Antimicrob Steward Healthc Epidemiol. 2026 Sep 29;6(1):e301. doi: 10.1017/ash.2026.10838 (PMC13760430; doi:10.1017/ash.2026.10838)
Supplement: Supplementary file 1 [file S2732494X26108389sup001.docx]

**SUPPLEMENTARY MATERIALS**

- **Index**
- **A summary of participant characteristics is presented in Table 1, while detailed participant characteristics are provided in Supplementary Table S1…….Page 2-3**
- **Supplementary Table S2. Verbatim quotations supporting qualitative themes ....... Page 4-14**
- **Supplementary Appendix S1. Semi-structured interview guide ............................. Page 15**
- **STROBE Statement—Checklist of items that should be included in reports of *cross-sectional studies* ….Page 16-17**
- **COREQ (COnsolidated criteria for REporting Qualitative research) Checklist…………………………………Page 18-19**

Supplementary Table S1: A summary of participant characteristics is presented in Table 1

| **Participant code** | **Gender / Age** | **Qualification/ (MDS Specialty)** | **Years of experience** | **Type of practice** | **Practice setting** |
| --- | --- | --- | --- | --- | --- |
| **P1** | F/32 | MDS (Endodontics and Conservative Dentistry) | 6 | Teaching and Clinical | Teaching institute |
| **P2** | M/59 | MDS (Prosthodontics and Crown and Bridge) | 30 | Teaching and Clinical | Teaching institute |
| **P3** | M/35 | MDS (Oral medicine and radiology) | 4 | Teaching and Clinical | Teaching institute |
| **P4** | M/34 | MDS (Oral medicine and radiology) | 4 | Teaching and Clinical | Teaching institute |
| **P5** | F/31 | BDS | 2 | Clinical | Private practice |
| **P6** | F/29 | BDS | 2 | Clinical | Private practice |
| **P7** | M/55 | BDS | 20 | Clinical | Private practice |
| **P8** | M/32 | MDS (Oral medicine and radiology) | 3 | Clinical | Private practice |
| **P9** | M/41 | MDS (Endodontics and Conservative Dentistry) | 10 | Clinical | Private practice |
| **P10** | F/43 | MDS (Periodontology and Implantology) | 12 | Clinical | PHC/CHC |
| **P11** | M/33 | BDS | 5 | Clinical | PHC/CHC |
| **P12** | M/36 | BDS | 7 | Clinical | Private practice |
| **P13** | M/50 | MDS (Oral medicine and radiology) | 22 | Teaching and Clinical | Teaching institute |
| **P14** | M/56 | MDS (Oral maxillofacial surgery) | 25 | Teaching and Clinical | Teaching institute |
| **P15** | M/38 | MDS (Endodontics and Conservative Dentistry) | 8 | Clinical | Private practice |
| **P16** | M/36 | BDS | 6 | Clinical | PHC/CHC |
| **P17** | M/41 | BDS | 9 | Clinical | PHC/CHC |
| **P18** | F/36 | BDS | 7 | Clinical | PHC/CHC |
| **P19** | M/48 | MDS (Prosthodontics and Crown and Bridge) | 16 | Teaching and Clinical | Teaching institute |
| **P20** | F/34 | MDS | 2 | Clinical | PHC/CHC |
| **P21** | F/37 | MDS (Oral medicine and radiology) | 5 | Teaching and Clinical | Teaching institute |
| **P22** | F/48 | MDS (Oral medicine and radiology) | 18 | Clinical | Private practice |
| **P23** | M/38 | BDS | 7 | Clinical | PHC/CHC |
| **P24** | M/41 | MDS (Endodontics and Conservative Dentistry) | 9 | Clinical | Private practice |
| **P25** | M/59 | MDS (Periodontology and Implantology) | 23 | Teaching and Clinical | Teaching institute |
| **P26** | M/39 | BDS | 4 | Clinical | PHC/CHC |
| **P27** | F/41 | MDS (Prosthodontics and Crown and Bridge) | 7 | Clinical | PHC/CHC |
| **P28** | M/38 | BDS | 3 | Clinical | PHC/CHC |
| **P29** | M/43 | MDS (Paediatric and preventive dentistry) | 13 | Teaching and Clinical | Teaching institute |
| **P30** | F/38 | MDS (Oral and maxillofacial surgery) | 5 | Clinical | Private practice |

**Supplementary Table S2: Verbatim quotations supporting qualitative themes**

| **Sno.** | **Theme** | **Subtheme** | **Quotes** |
| --- | --- | --- | --- |
| **1** | **Theme 1: The Knowledge – Practice interface** | **Experiential Perception of Resistance:** | *“AMR means that the antibiotic that we are giving, the specific bacteria on which it was working earlier, now it will not work. We know this when the patient is not getting relief, the swelling is not reducing, there is slow healing or persistent infection. Earlier the same antibiotics were working, but now it is not giving the same result.” (P2)*  *“If you have normal Amoxiclav then it is like a paracetamol in today’s time. If a person is having cough, then he is taking Amoxiclavs 625, Normally, what people do at home if someone is having any kind of disease then they have to take it. all the patients become resistant in a few days. They don’t even think which resistance is developing, they just want immediate relief.” (P16)*  *“In our area—like Uttar Pradesh, Bihar, Chhattisgarh and Jharkhand—around 60–70% of patients come from rural backgrounds. The main reason for this is the widespread practice of quackery. Often, a person who has worked as a compounder or in a medical store for three to four years goes to a village and opens a clinic. Patients go there and without any proper diagnosis, they are prescribed first-generation cephalosporins along with dexamethasone. (P26)*  *“Nowadays antibiotic resistance is increasing because antibiotics are being used very frequently, sometimes unnecessarily. Patients take antibiotics even for minor problems. Because of this frequent use, antibiotics are not working properly in dental infections also.” (P29)*  *“WHO has already said that AMR is among the top threats to human beings. It is a silent pandemic and nobody is aware or worried about it, but it is a very major threat.” (P5)*  *“Most of the patients take antibiotics on their own before coming to the dentist. They go directly to the medical store and purchase antibiotics without any prescription. By the time they come to us, the infection has already become complicated.” (P15)*  *“If you conduct a bacterial culture test in a normal individual, you will find that nearly 90% of the antibiotics show resistance. This is not just about one patient or one prescription—it shows how serious the problem has become at the community level. People want immediate pain relief, within minutes or hours and antibiotics are freely taken by them without thinking about what resistance is developing. Nobody is concerned about the long-term impact.” (P8)*  *“Earlier AMR was discussed only in books, but now we are facing it practically in daily routine practice. This shows how big the problem has become.” (P7)* |
|  |  | **Diffusion of Responsibility** | *“It is not that it is only in dentistry. In all the medical fraternities, dexamethasone and Amoxiclavs are run in such a combination. So, resistance is not because of dentists alone.” (P25)*  *“Most of the patients do not come in the beginning first they will try clove, salt water, oil pulling, or some powder suggested by relatives then they will take antibiotics by their own if it not gets healed then they come to dentist at that time they are already having sinus drain, swelling on the face and systemic involvement. At that time, we cannot treat immediately, so we prescribe antibiotics first to reduce the swelling and then we plan the treatment.” (P18)*  *“Because I am working in a teaching institute, many times I come across prescription slips from the ENT department or the medicine department. I have seen cases where three or four antibiotics are written together in a single prescription. So AMRis not happening only because of dentists; it is equally due to prescribing practices in the medical field as well.” (P13)*  *“The most common factor of AMR is the misuse of the antibiotics which is done in the society. Suppose the patient has no active infection and he is being regularly having antibiotics by his own, or by pharmacy, or by quacks, or by doctor also who knows; this is the most common cause.” (P14)*  *“Resistance is not only because of one doctor or one field. When antibiotics are misused everywhere—in villages, medical stores, hospitals—it becomes a public health problem, not an individual mistake.” (P1)* |
|  |  | **Habitual Normalization and Educational Gaps** | *“In undergraduate, we learn about antibiotics only in second year and that too just a little about resistance. Even in postgraduate training, there is no proper course that teaches how to actually prescribe antibiotics or how resistance develops in practice. Everything is theoretical. When you start clinical practice, you realise that you were not really prepared.” (P02)*  *“When we pass out, there is no proper training on how to prescribe antibiotics. In the beginning, we just follow what seniors are prescribing or what medical representatives suggest. That is how we start writing prescriptions. After some years, it becomes routine and then changing that practice becomes very difficult.” (P15)*  *“First of all, we do not have CMEs frequently. Even when CMEs happen, very rarely they are related to AMR or proper antibiotic prescribing. There are also hardly any workshops or seminars focused on this. Regular updates are needed because resistance patterns keep changing.” (P4)* |
| **2** | **Theme 2: Prescribing as a "Safety-measure":** | **Diagnostic Ambiguity and Resource Constraints** | *“The patient that we see regularly we don’t have adequate manpower and infrastructure and the kind of workload we have, sometimes it becomes difficult to treat the patient at that time we know very well that immediate treatment should be done but we don’t have time, manpower. Because of this, antibiotics are often prescribed to control the situation.” (P03)*  *“Sometimes patients come up with a lot of swelling and we can’t able to treat them at that moment until the swelling reduces. So, because of this, we prescribe antibiotics. Sometimes there are time constraints also, so that also becomes a reason to prescribe antibiotics.” (P5)*  *“If you have five hundred dentists practicing in your city, there will not be two such dentists who are doing culture sensitivity test. There is no sensitivity test, history is not taken properly, proper diagnosis is not done for the patient. We don’t even have the instruments or facilities. If you give culture, the report comes after two weeks or sometimes not at all.” (P24)*  *“As I am working in PHC we don’t have Culture sensitivity test kit on our table so it is not done routinely in dental practice even in medical also it is not done. We mostly depend on clinical signs and symptoms to decide the antibiotic because sending the patient for tests is not always feasible and even patients do not understand the importance of this also, they are not ready to wait and many times they cannot afford it so we end up giving them antibiotics.” (P11)*  *“By the time patients come to us, they have already taken some antibiotics from the medical store. They don’t give proper history and then it becomes difficult to judge what exactly is causing the infection.” (P20)*  *“We know that nearly 80% of the population in India lives in rural areas. Routine medical and dental checkups are not common in these areas, so patient is often unaware of whether they have any underlying systemic diseases or not Therefore as a precautionary measure, dentists prescribe antibiotics to prevent pre-operative and post-operative complications.” (P10)* |
|  |  | **Risk Avoidance and Defensive Practice** | *In India, we cannot always rely on patients to maintain proper oral hygiene. Even if treatment is provided and oral hygiene instructions are given, many patients do not follow them. Once they leave the clinic, they may consume tobacco products or fail to brush their teeth properly. (P23)*  *“For a particular extraction also, we keep the patient on antibiotics for ten to twelve days, sometimes for 10 days. This is done because we don’t want any post-operative complications. If something happens later, then the patient will blame the doctor.” (P19)*  *“In medically compromised patients like diabetics or elderly patients, we prefer to give antibiotics because there is always a fear of infection or delayed healing. We cannot take a chance with such patients.” (P27)*  *“When the patient is medically compromised, you cannot experiment. Even if the infection looks localised, we give antibiotics to prevent any systemic spread.” (P4)*  *“If there is a lot of systemic involvement or facial swelling, then we prescribe TDS. If it is visible only in X-ray and there is no swelling, then we prescribe BD. It depends on the condition because we don’t want it to worsen.” (P4)* |
| **3** | **Theme 3: Socio-Cultural influences on Antibiotic Prescribing** | **"Prescription as Satisfaction" Trap** | *“Most of the patients come with the mindset that if pain is there, antibiotic is compulsory. They don’t want to listen to explanations about treatment or infection. They only want a tablet that will reduce pain quickly. If we don’t prescribe antibiotics, they feel dissatisfied and sometimes even argue, saying that earlier doctors always gave them antibiotics.” (P14)*  *“Patients feel that antibiotics are necessary for pain relief. If antibiotics are not prescribed, they think that proper treatment has not been done. They don’t feel satisfied unless some medicine is written.” (P06)*  *“When we try to explain that antibiotics are not required, patients feel we are wasting their time. They say, ‘Just give me medicine, doctor, I will manage later.’ Because of this pressure, sometimes antibiotics are prescribed.” (P07)*  *“Many patients come and say that earlier also when they had pain, they were given antibiotics and it worked. So now they expect the same thing. If we say that antibiotics are not needed, they feel that we are not treating them properly.” (P13)*  *“The patient will say, sir give me such a medicine that I should get rid of my pain within one day. They don’t care which resistance is developing, what treatment is needed, is it safe to take antibiotic or not. They just want immediate relief.” (P15)*  *“Many times, patients come only for medicine. They don’t want extraction or RCT on that day. They say, ‘Today just give tablets, I will come later.’ In such cases, to satisfy the patient and reduce pain, antibiotics are given even though we know treatment is more important.” (P18)* |
|  |  | **Normalization and Influencing Norms** | *“When you see that everyone around you is prescribing antibiotics for similar cases, it becomes a normal thing. You don’t feel that you are doing something wrong because it is the routine practice everywhere.” (P20)*  *“There are no specific national dental guidelines, so usually we depend on what seniors are doing or what we have seen in practice. That becomes the reference for prescribing.” (P21)*  *“Medical representatives regularly come and explain about new antibiotics or combinations. After hearing them repeatedly, it stays in your mind and you tend to prescribe those medicines more confidently.” (P06)*  *“Sometimes we do get influenced by medical representatives because many times we are not aware of new drugs or new combinations coming into the market. There are no regular updates or guidelines, so whatever information we get is mostly from them. At the same time, we don’t blindly follow what they say. If a medicine is suggested, we usually go back, read about it, check the literature and then decide. Finally, we prescribe only those medicines which we feel are effective and safe based on our own understanding and experience.” (P04)*  *“Usually, the medical representatives promote the same antibiotics that are already being prescribed commonly. So, it just strengthens the idea that these medicines are safe and effective for most cases.” (P14)*  *“I never prefer any medicine or brand which I don’t know. I prescribe based on my own experience and understanding of the disease, not because someone else is prescribing it or because it is promoted.” (P15)*  *“Patients get information from Google, YouTube and social media and they come asking for specific antibiotics which they think will work. They already have a medicine name in mind when they come.” (P17)*  *“Social media also influences. Patients search which medicine will work if there is swelling in the teeth. It is written there that amoxicillin will work, so they take it on their own without consulting.” (P2)*  *“Sometimes patients argue and say, ‘I read on the internet that this antibiotic is best.’ They don’t understand that every case is different and it becomes difficult to convince them otherwise.” (P09)*  *“Social media is a different thing, print media and mass media are different things. It should come through newspapers or news channels or peer-reviewed magazines. Social media like Facebook or Instagram should not be the source.” (P14)* |
| **4** | **Theme 4: System-Level Barriers and the Need for Stewardship Reform** | **The "Guideline Gap** | *“There is no protocol as such that we follow. We prescribe according to the patient and the condition. There is no specific guideline that tells us what to give, for how long, or in which situation.” (P04)*  *“Foreign guidelines are made for their own population. There people are more educated and concerned about their health and India is a very diverse and developing country. Many patients are not well educated and there is lots of cultural beliefs also. So, we cannot apply those guidelines directly to Indian patients.” (P18)*  *“In Western countries, patients come early and follow instructions. Here, patients come very late, after taking medicines on their own and many times they don’t return for follow-up. So, guidelines made for other countries don’t fit our situation.” (P22)*  *“India is not one kind of population. Some patients understand, some don’t. Some can come for follow-up, some cannot. So, one guideline cannot work the same way for all patients.” (P06)*  *“Most guidelines assume that diagnostic facilities are available and that patients can be reviewed. But in many places, we don’t even have basic diagnostics and patients may not come back. So practically, those guidelines are difficult to follow.” (P26)*  *“For the same condition, different dentists prescribe different antibiotics and for different durations. It depends completely on the dentist because there is no common guideline to follow.” (P15)*  *“If there were clear Indian guidelines, we could confidently avoid unnecessary antibiotics. But since there is no proper direction, many times antibiotics are given to be on the safer side.” (P22)* |
|  |  | **Unregulated Access and Regulatory Gaps** | *“Over the counter is definitely a great threat because in our country there is no standard guideline regarding the drug prescription and the medications to be purchased by the patients. Without any prescription none of the medical supplier should give a medication, but this is not followed.” (P17)*  *“In India, buying antibiotics has become like buying items from a grocery store. Just like we go to a shop and purchase whatever we need, people go to the pharmacy and ask for antibiotics and they get it without any prescription. Even though there are rules that antibiotics should not be given like this, nobody follows them. Because of this easy availability, patients misuse antibiotics, resistance increases and finally doctors have to manage all the complications.” (P08)*  *“Antibiotics are easily available in medical stores without prescription. Patients take them for pain or swelling and then come to us when it doesn’t work. By that time, the infection has already become complicated.” (P23)*  *“Most of the patients take antibiotics on their own before coming to the dentist. They go directly to the medical store and purchase antibiotics without any prescription. This misuse is happening very commonly now.” (P07)*  *“We already have policies that antibiotics should not be given without prescription, but in practice this is not followed. Because of this, resistance is increasing and doctors are left to deal with the complications.” (P15)*  *“Because patients have already taken antibiotics from outside, we don’t know what they have taken or for how many days. This makes managing the infection more difficult.” (P22)* |
|  |  | **Readiness for Stewardship and Oversight** | *“As in teaching institute with the number of patients that we see regularly, we don’t have adequate manpower and infrastructure. Because of the workload, sometimes it becomes difficult to treat the patient immediately. Even though we know that definitive treatment should be done, antibiotics are often prescribed to control the situation.” (P01)*  *“In PHC settings, even though the patient load is not always high, we often face shortages of materials, funding and basic infrastructure. Facilities such as dental chairs or OPG machines are sometimes not available and there are also limitations in the number of doctors. When patients present with caries or dental pain, we do not have facilities for taking radiographs, so we either prescribe antibiotics or refer them to private setups for X-rays. Because of these constraints, antibiotics are frequently prescribed.” (P17)*  *“In OPD settings, we have very limited time for each patient. Explaining everything, doing investigations and completing treatment on the same day is not always possible. So, antibiotics become a temporary solution.” (P18)*  *“When there are many patients waiting, it becomes difficult to give sufficient time to each case. Antibiotics help in reducing symptoms so that the patient can come back later for treatment.” (P01)*  *“Culture and sensitivity tests are not done routinely in dental practice. We mostly depend on clinical signs and symptoms to decide the antibiotic because sending the patient for tests is not always feasible. We don’t even have proper instruments or facilities for culture tests. Even if samples are sent, many times reports don’t come/delayed.” (P10)*  *“In government setups, there is an online record system where you can see what course the patient has taken. But in private practice, there is nothing like that, so mistakes keep repeating.” (P5)*  *“In private dental practice, there is absolutely no monitoring system. Nobody keeps an eye on how many antibiotics are prescribed or for what reason. There is no audit, no review and no feedback. So whatever pattern a dentist follows continues for years without anyone questioning it.” (P29)*  *“Audit will actually be helpful. It is not about blaming anyone or pointing fingers. If someone looks at our prescriptions and makes us think about why we are prescribing, it will automatically improve practice.” (P13)*  *“If prescriptions are reviewed and proper feedback is given, it will really help dentists improve. Just making rules or restrictions without explaining the reason will not work in practice.” (P22)*  *“We are definitely overusing antibiotics. If this continues, then in the future even simple infections will not respond. We are already seeing resistance and it will only get worse.” (P29)*  *“Sometimes after prescribing, I start thinking—was it really needed or not? Earlier this thought did not come, but now it is coming more often.” (P5)*  *“There should be clear guidelines for dentists. If there is a standard protocol, then everyone will follow it and misuse will automatically reduce.” (P15)*  *“OTC sale should be stopped completely. Without prescription, no antibiotic should be given. Otherwise, whatever we do inside the clinic will not make much difference.” (P14)*  *“For the general population, awareness should be given through newspapers and news channels. People should understand that medicines should not be taken without prescription.” (P14)* |

**Supplementary Appendix S1. Semi-structured interview guide**

| Sr no. | Questions |
| --- | --- |
| 1 | Can you describe your routine dental practice and the types of clinical situations in which you usually prescribe antibiotics? Which procedures or conditions most often lead to antibiotic prescriptions in your experience? |
| 2 | How often do you prescribe antibiotics in a typical week, and which antibiotics do you most commonly use? What factors influence your choice, including dosage and duration? |
| 3 | As a practising dentist, how do you interpret the concept of antibiotic resistance, especially in situations where infections do not respond as expected to standard treatments? |
| 4 | From your clinical experience, what factors do you think contribute to the development of antibiotic resistance in dental settings? *Probes:* unnecessary use, repeated prescriptions, self-medication, incomplete courses, lack of new drugs. |
| 5 | Have you observed any changes over time in antibiotic effectiveness or treatment outcomes, such as slow healing or persistent infections? Can you describe any case that influenced your perception? |
| 6 | What factors usually influence your decision to prescribe antibiotics for dental pain or infection? *Probes:* patient expectations, diagnostic uncertainty, time constraints, availability of investigations, fear of complications. |
| 7 | Can you recall a situation where a patient strongly demanded antibiotics even when clinical signs did not justify it? How did you manage that situation, and what influenced your final decision? |
| 8 | When you decide to prescribe antibiotics, how do clinical guidelines influence your decision-making? Are there situations where you need to adjust dosage or treatment plans based on patient-related factors? |
| 9 | Can you describe any experience that made you realise the seriousness of antimicrobial resistance in your practice? Did that experience change your prescribing approach or highlight any need for additional training? |
| 10 | What are your views on strategies to improve antibiotic prescribing in dentistry, such as training programs, stewardship initiatives, or prescription audits? How do you think these could be effectively implemented? |

**STROBE Statement—Checklist of items that should be included in reports of *cross-sectional studies***

|  | Item No | Recommendation | Page no. |
| --- | --- | --- | --- |
| **Title and abstract** | 1 | (*a*) Indicate the study’s design with a commonly used term in the title or the abstract | 3 |
|  |  | (*b*) Provide in the abstract an informative and balanced summary of what was done and what was found | 3 |
| Introduction | | |  |
| Background/rationale | 2 | Explain the scientific background and rationale for the investigation being reported | 4-5 |
| Objectives | 3 | State specific objectives, including any prespecified hypotheses | 4-5 |
| Methods | | |  |
| Study design | 4 | Present key elements of study design early in the paper | 4-7 |
| Setting | 5 | Describe the setting, locations, and relevant dates, including periods of recruitment, exposure, follow-up, and data collection | 4-7 |
| Participants | 6 | (*a*) Give the eligibility criteria, and the sources and methods of selection of participants | 4-7 |
| Variables | 7 | Clearly define all outcomes, exposures, predictors, potential confounders, and effect modifiers. Give diagnostic criteria, if applicable | 4-7 |
| Data sources/ measurement | 8* | For each variable of interest, give sources of data and details of methods of assessment (measurement). Describe comparability of assessment methods if there is more than one group | *4-7* |
| Bias | 9 | Describe any efforts to address potential sources of bias | 4-7 |
| Study size | 10 | Explain how the study size was arrived at | 4-7 |
| Quantitative variables | 11 | Explain how quantitative variables were handled in the analyses. If applicable, describe which groupings were chosen and why | 4-7 |
| Statistical methods | 12 | (*a*) Describe all statistical methods, including those used to control for confounding | 7 |
|  |  | (*b*) Describe any methods used to examine subgroups and interactions | NA |
|  |  | (*c*) Explain how missing data were addressed | 7 |
|  |  | (*d*) If applicable, describe analytical methods taking account of sampling strategy | 6 |
|  |  | (*e*) Describe any sensitivity analyses | NA |
| Results | | |  |
| Participants | 13* | (a) Report numbers of individuals at each stage of study—eg numbers potentially eligible, examined for eligibility, confirmed eligible, included in the study, completing follow-up, and analysed | 7 |
|  |  | (b) Give reasons for non-participation at each stage | 7 |
|  |  | (c) Consider use of a flow diagram | NA |
| Descriptive data | 14* | (a) Give characteristics of study participants (eg demographic, clinical, social) and information on exposures and potential confounders | 7 |
|  |  | (b) Indicate number of participants with missing data for each variable of interest | 7 |
| Outcome data | 15* | Report numbers of outcome events or summary measures | 7 |
| Main results | 16 | (*a*) Give unadjusted estimates and, if applicable, confounder-adjusted estimates and their precision (eg, 95% confidence interval). Make clear which confounders were adjusted for and why they were included | 7 |
|  |  | (*b*) Report category boundaries when continuous variables were categorized | 7 |
|  |  | (*c*) If relevant, consider translating estimates of relative risk into absolute risk for a meaningful time period | NA |
| Other analyses | 17 | Report other analyses done—eg analyses of subgroups and interactions, and sensitivity analyses | NA |
| Discussion | | |  |
| Key results | 18 | Summarise key results with reference to study objectives | 7 |
| Limitations | 19 | Discuss limitations of the study, taking into account sources of potential bias or imprecision. Discuss both direction and magnitude of any potential bias | 13 |
| Interpretation | 20 | Give a cautious overall interpretation of results considering objectives, limitations, multiplicity of analyses, results from similar studies, and other relevant evidence | 11-13 |
| Generalisability | 21 | Discuss the generalisability (external validity) of the study results | 13 |
| Other information | | |  |
| Funding | 22 | Give the source of funding and the role of the funders for the present study and, if applicable, for the original study on which the present article is based | 14 |

*Give information separately for exposed and unexposed groups.

**Note:** An Explanation and Elaboration article discusses each checklist item and gives methodological background and published examples of transparent reporting. The STROBE checklist is best used in conjunction with this article (freely available on the Web sites of PLoS Medicine at http://www.plosmedicine.org/, Annals of Internal Medicine at http://www.annals.org/, and Epidemiology at http://www.epidem.com/). Information on the STROBE Initiative is available at www.strobe-statement.org.

**COREQ (COnsolidated criteria for REporting Qualitative research) Checklist**

A checklist of items that should be included in reports of qualitative research. You must report the page number in your manuscript where you consider each of the items listed in this checklist. If you have not included this information, either revise your manuscript accordingly before submitting or note N/A.

| **Topic** | **Item No.** | **Guide Questions/Description** | **Reported on**  **Page No.** |
| --- | --- | --- | --- |
| **Domain 1: Research team**  **and reflexivity** | | | |
| *Personal characteristics* | | | |
| Interviewer/facilitator | 1 | Which author/s conducted the interview or focus group? | 6 |
| Credentials | 2 | What were the researcher’s credentials? E.g. PhD, MD | 6 |
| Occupation | 3 | What was their occupation at the time of the study? | 6 |
| Gender | 4 | Was the researcher male or female? | 6 |
| Experience and training | 5 | What experience or training did the researcher have? | 6 |
| *Relationship with*  *participants* | | | |
| Relationship established | 6 | Was a relationship established prior to study commencement? | 6 |
| Participant knowledge of  the interviewer | 7 | What did the participants know about the researcher? e.g. personal  goals, reasons for doing the research |  |
|  |  |  | 6 |
|  |  |  |  |
| Interviewer characteristics | 8 | What characteristics were reported about the inter viewer/facilitator?  e.g. Bias, assumptions, reasons and interests in the research topic |  |
|  |  |  | 6 |
|  |  |  |  |
| **Domain 2: Study design** | | | |
| *Theoretical framework* | | | |
| Methodological orientation and Theory | 9 | What methodological orientation was stated to underpin the study? e.g. grounded theory, discourse analysis, ethnography, phenomenology,  content analysis |  |
|  |  |  | 6 |
|  |  |  |  |
| *Participant selection* | | | |
| Sampling | 10 | How were participants selected? e.g. purposive, convenience,  consecutive, snowball |  |
|  |  |  | 6 |
|  |  |  |  |
| Method of approach | 11 | How were participants approached? e.g. face-to-face, telephone, mail,  email |  |
|  |  |  | 6 |
|  |  |  |  |
| Sample size | 12 | How many participants were in the study? | 6 |
| Non-participation | 13 | How many people refused to participate or dropped out? Reasons? | NA |
| *Setting* | | | |
| Setting of data collection | 14 | Where was the data collected? e.g. home, clinic, workplace | 6 |
| Presence of non-  participants | 15 | Was anyone else present besides the participants and researchers? |  |
|  |  |  | 6-7 |
|  |  |  |  |
| Description of sample | 16 | What are the important characteristics of the sample? e.g. demographic  data, date |  |
|  |  |  | 6 |
|  |  |  |  |
| *Data collection* | | | |
| Interview guide | 17 | Were questions, prompts, guides provided by the authors? Was it pilot  tested? | 6 |
|  |  |  |  |
| Repeat interviews | 18 | Were repeat inter views carried out? If yes, how many? | NA |
| Audio/visual recording | 19 | Did the research use audio or visual recording to collect the data? | 6 |
| Field notes | 20 | Were field notes made during and/or after the inter view or focus group? | 6-7 |
| Duration | 21 | What was the duration of the inter views or focus group? | 6 |
| Data saturation | 22 | Was data saturation discussed? | 6-7 |
| Transcripts returned | 23 | Were transcripts returned to participants for comment and/or correction? | NA |
| **Domain 3: analysis and**  **findings** |  |  |  |
| *Data analysis* |  |  |  |
| Number of data coders | 24 | How many data coders coded the data? | 7 |
| Description of the coding  tree | 25 | Did authors provide a description of the coding tree? | 7 |
| Derivation of themes | 26 | Were themes identified in advance or derived from the data? | 7 |
| Software | 27 | What software, if applicable, was used to manage the data? | 7 |
| Participant checking | 28 | Did participants provide feedback on the findings? | NA |
| *Reporting* |  |  |  |
| Quotations presented | 29 | Were participant quotations presented to illustrate the themes/findings?  Was each quotation identified? e.g. participant number | 7-11 |
| Data and findings consistent | 30 | Was there consistency between the data presented and the findings? | 7-11 |
| Clarity of major themes | 31 | Were major themes clearly presented in the findings? | 7-11 |
| Clarity of minor themes | 32 | Is there a description of diverse cases or discussion of minor themes? | 7-11 |

Developed from: Tong A, Sainsbury P, Craig J. Consolidated criteria for reporting qualitative research (COREQ): a 32-item checklist for interviews and focus groups. *International Journal for Quality in Health Care*. 2007. Volume 19, Number 6: pp. 349 – 35
